# Genome-wide exploration and characterization of miR172/*euAP2* genes in *Brassica napus* L. for likely role in flower organ development

**DOI:** 10.1186/s12870-019-1936-2

**Published:** 2019-08-01

**Authors:** Tengyue Wang, Xiaoke Ping, Yanru Cao, Hongju Jian, Yumin Gao, Jia Wang, Yingchao Tan, Xinfu Xu, Kun Lu, Jiana Li, Liezhao Liu

**Affiliations:** College of Agronomy and Biotechnology, Chongqing Engineering Research Center for Rapeseed, Academy of Agricultural Sciences, State Cultivation Base of Crop Stress Biology for Southern Mountainous Land, Southwest University, Chongqing, China

**Keywords:** *Brassica napus*, miR172, *euAP2*, Evolution, Expression analysis

## Abstract

**Background:**

*APETALA2*-like genes encode plant-specific transcription factors, some of which possess one microRNA172 (miR172) binding site. The miR172 and its target *euAP2* genes are involved in the process of phase transformation and flower organ development in many plants. However, the roles of miR172 and its target *AP2* genes remain largely unknown in *Brassica napus* (*B. napus*).

**Results:**

In this study, 19 *euAP2* and four miR172 genes were identified in the *B. napus* genome. A sequence analysis suggested that 17 *euAP2* genes were targeted by Bna-miR172 in the 3′ coding region. *EuAP2*s were classified into five major groups in *B.napus*. This classification was consistent with the exon-intron structure and motif organization. An analysis of the nonsynonymous and synonymous substitution rates revealed that the *euAP2* genes had gone through purifying selection. Whole genome duplication (WGD) or segmental duplication events played a major role in the expansion of the *euAP2* gene family. A cis-regulatory element (CRE) analysis suggested that the *euAP2*s were involved in the response to light, hormones, stress, and developmental processes including circadian control, endosperm and meristem expression. Expression analysis of the miR172-targeted *euAP2s* in nine different tissues showed diverse spatiotemporal expression patterns. Most *euAP2* genes were highly expressed in the floral organs, suggesting their specific functions in flower development. *BnaAP2–1*, *BnaAP2–5* and *BnaTOE1–2* had higher expression levels in late-flowering material than early-flowering material based on RNA-seq and qRT-PCR, indicating that they may act as floral suppressors.

**Conclusions:**

Overall, analyses of the evolution, structure, tissue specificity and expression of the *euAP2* genes were peformed in *B.napus*. Based on the RNA-seq and experimental data, *euAP2* may be involved in flower development. Three *euAP2* genes (*BnaAP2–1*, *BnaAP2–5* and *BnaTOE1–2*) might be regarded as floral suppressors. The results of this study provide insights for further functional characterization of the miR172 /*euAP2* module in *B.napus*.

**Electronic supplementary material:**

The online version of this article (10.1186/s12870-019-1936-2) contains supplementary material, which is available to authorized users.

## Background

MicroRNAs are a class of endogenous, small non-coding RNAs (20–24 nucleotides) with critical roles in the post-transcriptional regulation of gene expression [1]. Plant miRNAs have been reported to be involved in many biological processes, such as auxin signalling [2], flowering time regulation [3], leaf development [4] and stress responses [5, 6]. Recent studies have also indicated that miRNAs can regulate developmental timing and floral induction in a variety of plants via their corresponding target genes [7–13]. In particular, two highly conserved microRNAs, namely, miR156 and miR172, have been identified as master regulators of vegetative phase change [14–19].

MiR172 was first obtained by small-RNA sequencing in Arabidopsis and has been found in many plants because of its high conservation [20]. The miR172 family is a major component of the age pathway, and it is encoded by MIR172a-e genes in Arabidopsis. The target genes of this family in Arabidopsis include six *APETALA-2 (AP2)*-type genes: *APETALA2* (*AP2*), *TARGET OF EAT 1* (*TOE1*, *TOE2*, and *TOE*3), *SCHLAFMUTZE (SMZ)* and *SCHNARCHZAPFEN (SNZ)*. *AP2* is involved in floral organ formation, and the other five AP2-like genes are mainly floral suppressors [3, 21, 22]. The AP2 proteins have one or more AP2 domains consisting of 60–70 highly conserved amino acids (aa) and two conserved components, namely, the YRG and RAYDmotifs, at the N- and C-terminus of the AP2 domain respectively [23–25].

MiR172 and its target genes play key roles in flowering time and floral organ differentiation. In Arabidopsis, overexpressing all the miR172 target genes except *TOE3* led to delayed flowering [18, 22, 24]. In contrast, the *toe1toe2* double mutant, *toe1toe2smzsnz* quadruple mutant and *toe1toe2toe3smzsnzap2* sextuple mutant all showed early flowering, and the flowering time advanced as the number of mutant genes increased [26, 27]. On the one hand, this result showed functional redundancy among the *AP2* genes; on the other hand, it confirmed that *AP2* genes negatively regulate flowering time in Arabidopsis. In the maize mutant *glossy15* (*gl15*), young leaves showed premature adult leaf characteristics because miR172-targeted *GL15* played a role in the juvenile to adult transition [28]. Overexpressing miR172 not only accelerated flowering but also resulted in abnormal flower development in Arabidopsis including a loss of petals and homologous transformation of sepals to carpels [3, 22, 26], the phenotype similar to the *ap2* mutant. When a 6-base mutation was introduced at the miR172 binding site of *AP2* to produce a miR172 resistance gene, a large number of petals were generated and the stamens were degenerated [22]. Overexpression of miR172b in rice delayed the transition from spikelet meristem to floral meristem and resulted in homeotic transformation in floral organs [29]. Overexpression of miR172 in tobacco also resulted in the conversion of sepals to petals, while *35S::AP2m3* plants exhibited floral patterning defects that included the proliferation of numerous petals, stamens and carpels [30]. In barley, *Cleistogamy1* (Cly1) replaces the synonymous nucleotides at the miR172 target site and leads to the failure of flowering and a closed-fertilization phenotype [31].

*Brassica napus* is the second largest oil crop in the world. This species is an allopolyploid (AACC, 2n = 4x = 38) that evolved from a natural interspecies cross between *Brassica rapa* (genome AA, 2n = 20) and *Brassica oleracea* (genome CC, 2n = 18) [32]. Flowering represents the transition from the vegetative stage to the reproductive stage. Flowering appropriately is crucial to ensure reproductive success and thus determines the growth period, yield and seed quality in *B. napus*. Despite the roles of *AP2* genes in Arabidopsis has been well described, the roles of miR172 and its target *AP2* genes in determining flowering and floral development are still unclear in *B. napus*. Although the MiRNA172c-APETALA2–1 node has been identified as a key regulator of nitrogen fixation in common bean [33], the interaction between the *AP2* and MiR172 in *B.napus* has not clearly understood. Therefore, it would be intriguing to perform a genome-wide functional analysis of miR172/*euAP2* in *B.napus*. In this study, we systematically analysed the chromosomal location, evolutionary relationships, duplication events, collinearity, gene structure, conserved motifs and upstream cis-elements among miR172 members and their *euAP2* target genes. RNA sequencing (RNA-seq) and quantitative real-time PCR (qRT-PCR) were also carried out to analyse the expression pattern of *euAP2s* in diverse tissues and developmental stages.

## Results

### Identification of *euAP2* and miR172 genes in *B. napus*

Eight characterized AP2 protein sequences in Arabidopsis were used as the query sequences to identify the *euAP2* gene family in the *B. napus* genome using BLASTP searches with an E-value of ≤10^− 10^, and 19 putative *euAP2* family genes were identified. Then, the SMART, Pfam, CDD and HMMER databases were used to verify the AP2 domains, and all 19 predicted protein had one or two AP2 domains. As shown in Table 1, we numbered the *B. napus euAP2* according to their closest Arabidopsis homolog.

**Table 1:** List of putative *euAP2* genes and their protein features in *B.napus*

Gene id	Gene name	Gene id	Gene name	Chromosome	Target_start	Target_end	Amino acid length (aa)	pI	MW	Subcellular location	Conserved AP2 motif number
AT4G36920	*AthAP2*	BnaA01g34730D	*BnaAP2–1*	A01_random	581	601	233	8.89	25769.56	Nucleus	1
		BnaA03g53830D	*BnaAP2–2*	A03	1344	1364	432	6.77	47937.77	Nucleus	2
		BnaC01g01710D	*BnaAP2–3*	C01	–	–	400	6.02	44092.70	Nucleus	2
		BnaCnng39690D	*BnaAP2–4*	Cnn_random	1342	1362	431	6.41	47685.41	Nucleus	2
		BnaCnng71740D	*BnaAP2–5*	Cnn_random	578	598	227	7.87	25015.64	Nucleus	1
AT3G54990	*AthSMZ*	BnaA09g54740D	*BnaSMZ-1*	A09_random	953	973	343	9.42	38181.8	Cytoplasm	1
		BnaC06g15540D	*BnaSMZ-2*	C06	911	931	328	9.15	36790.23	Cytoplasm	1
		BnaC08g25840D	*BnaSMZ-3*	C08	956	976	344	9.39	38334.04	Cytoplasm	1
AT2G39250	*AthSNZ*	BnaC02g15640D	*BnaSNZ*	C02	–	–	128	6.27	14495.41	Cytoplasm	1
AT2G28550	*AthTOE1*	BnaA03g22100D	*BnaTOE1–1*	A03	1460	1480	436	6.51	47782.51	Cytoplasm. Nucleus	2
		BnaA07g13990D	*BnaTOE1–2*	A07	1512	1532	454	6.48	49570.20	Nucleus	2
		BnaC03g26480D	*BnaTOE1–3*	C03	1384	1404	417	6.61	45569.96	Cytoplasm. Nucleus	1
		BnaC04g15640D	*BnaTOE1–4*	C04	1496	1516	454	6.48	49308.84	Nucleus	2
		BnaC09g13430D	*BnaTOE1–5*	C09	694	714	103	8.09	11002.98	Nucleus	1
AT5G60120	*AthTOE2*	BnaA02g06490D	*BnaTOE2–1*	A02	1184	1204	431	8.56	47492.05	Cytoplasm. Nucleus	1
		BnaA10g12950D	*BnaTOE2–2*	A10	1412	1432	452	7.74	49305.81	Cytoplasm. Nucleus	1
		BnaC09g35430D	*BnaTOE2–3*	C09	1187	1207	454	7.74	49632.26	Cytoplasm. Nucleus	1
AT5G67180	*AthTOE3*	BnaA07g12050D	*BnaTOE3–1*	A07	1315	1335	360	8.91	40692.38	Nucleus	2
		BnaC07g16190D	*BnaTOE3–2*	C07	1272	1292	361	9.11	40835.46	Cytoplasm. Nucleus	2

The chromosome location, protein sequence length, molecular weight (MW), isoelectric point (pI), and subcellular localization were analysed. The size of the euAP2 proteins ranged from 103 (BnaTOE1–5) to 454 (BnaTOE2–3) amino acids. The predicted Mw of these deduced proteins varied from 11.00 kDa to 49.63 kDa, and the PI values varied from 6.02 to 9.42. The predicted subcellular localization results showed that nine euAP2 proteins were located in the nuclear region, four proteins were located in the cytoplasm region and the other six proteins were located in the nuclear and cytoplasm regions. These results indicate that different euAP2 family proteins may perform different functions.

Four putative miR172 family members (Bna-miR172a, Bna-miR172b, Bna-miR172c, and Bna-miR172d) in *B. napus* were predicted in miRbase, their precursor and mature sequences were shown in Additional file 1: Table S1. Multiple sequence alignments of the miR172 precursor and mature sequences were shown in Additional file 8: Figure S1a, b. The four mature miR172 sequences were highly conserved except for the first base, whereas divergence was observed in the precursor sequences. The secondary structure of the Bna-MIRl72 gene transcript was predicted by Mfold (Additional file 8: Figure S1c), and the four precursor sequences formed different stem-loop structures, which may lead to functional differences among members.

**Fig. 1:**
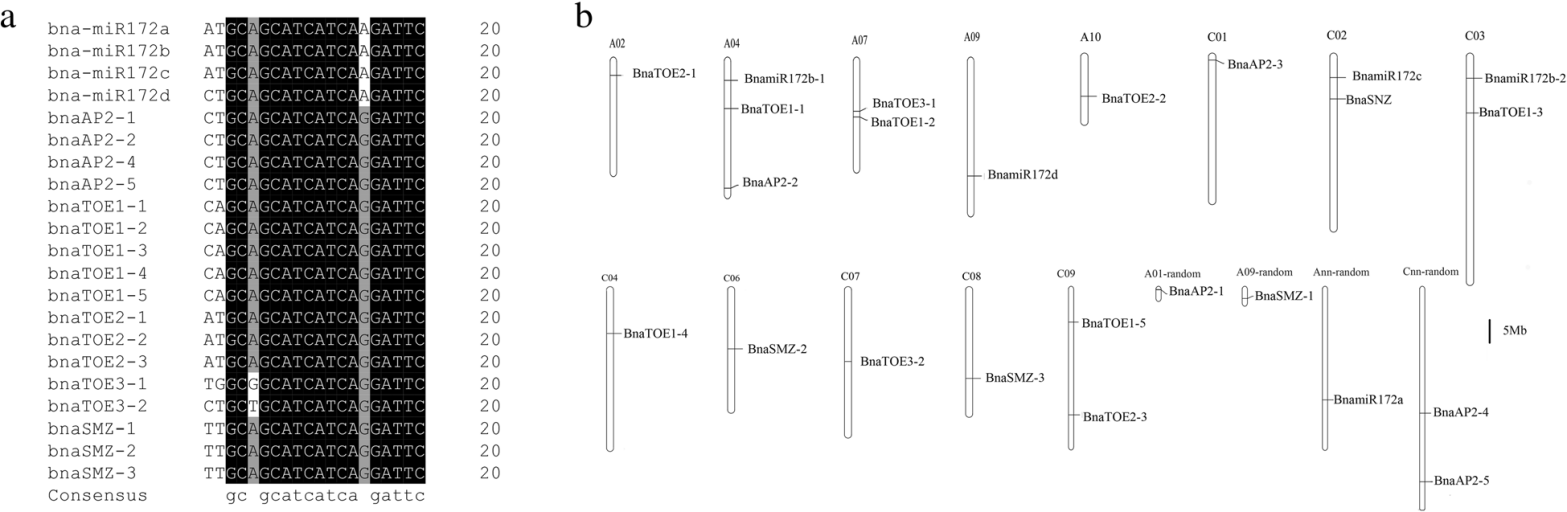
Multiple sequence alignment and chromosomal localization of miR172 and *euAP2 in B.napus.* **a** Alignment of the Bna-miR172 mature sequence compared with *euAP2* genes; **b** Chromosomal location of *euAP2*s and the miR172 precursor sequence in the *B. napus* genome

Target prediction for miRNAs is based on the high degree of homology between miRNAs and their target genes in plants [34]. Two target prediction servers, namely, TAPIR and psRNATarget, were used to identify potential targets of miR172 in *B. napus*. A total of 17 targets carrying the miRNA response element (MRE) for miR172 were predicted, and the position of miRNA binding sites were displayed in Table 1. A comparison of the Bna-miR172 mature sequences to the *euAP2* sequences showed that 17 *euAP2*s contained sequences complementary to the Bna-miR172 mature sequences, with one to three base mismatches (Fig. 1a). All the identified target sites of Bna-miR172 were located in the coding regions of *euAP2* genes.

The chromosome distribution of the *euAP2* genes and Bna-miR172 precursors is shown in Fig. 1b. The 19 *euAP2s* and four Bna-miR172s were located in 13 known chromosomes, and four were located in the scaffolds. These genes and miRNA precursors were unevenly distributed in the *B .napus* sub-genomes, with six and nine *euAP2* genes located in the A and C genomes, respectively. The other four *euAP2* genes mapped onto A01-random, A09-random, Ann-random and Cnn-random. The Bna-miR172 genes were distributed in A03, A09, C02, C03 and Ann_random. However, no *euAP2* genes and Bna-miR172 precursors were located on A01, A04, A05, A06, A08, and C01 chromosomes. The absence of genes on the chromosomes and uneven distribution on the sub-genomes of *euAP2* genes clarified their translocation during evolution.

### Phylogenetic and selection pressure analysis of the *euAP2* and Bna-miR172 genes

**Fig. 2:**
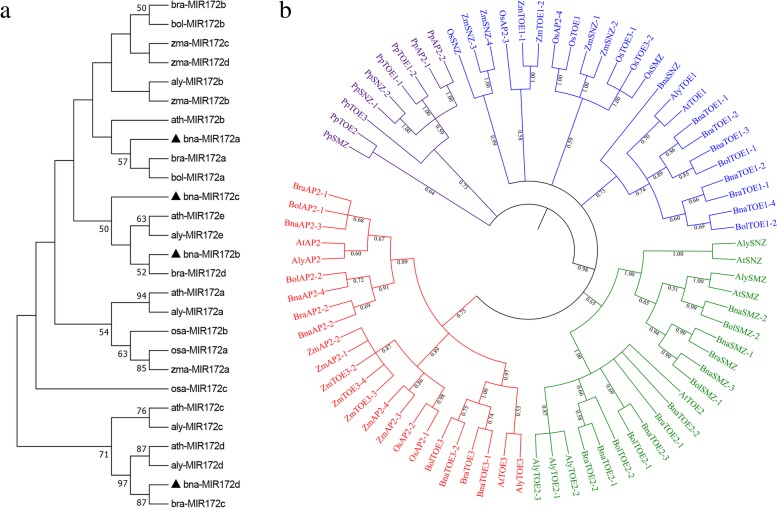
Phylogenetic tree of miR172 and *AP2*-type genes in *B. napus* and other seven species. The different-coloured arcs indicate different subgroups of *euAP2* genes. **a** Phylogenetic relationship of miR172  genes; **b**. Phylogenetic relationship of *AP2 *genes. Numbers on the branches are the bootstrap percentage values based on 1000 replicates, and only values of > 50% are shown. *Bna*: *Brassica napus, Ath*: Arabidopsis*, Bol*: *Brassica oleracea, Bra*: *Brassica rapa, Aly: Arabidopsis lyrata*, *Pp: Physcomitrella patens, Os: Oryza sativa* and *Zm: Zea mays*

To evaluate the evolutionary relationships of the * AP2* and miR172 gene members, an unrooted neighbour-joining phylogenetic tree was created using the precursor sequence of miR172 and amino acid sequences of *AP2* genes from Arabidopsis, *B. napus*, *B. rapa*, *B. oleracea*, rice, maize, moss and *Arabidopsis lyrata*. However, no miR172 gene was identified in moss. MIR172 genes from other seven plant species could be classified into four groups according to the phylogenetic analysis. Every group had at least one MIR172 gene from Arabidopsis (Fig. 2a). The AP2 genes from the eight species were assigned to four groups according to the phylogenetic topology (Fig. 2b). The *BnaAP2* genes were firstly clustered with *AP2*s from two progenitors of *B. napus*, followed by Arabidopsis and *A. lyrata*, which formed a specific Brassicaceae clade. EuAP2 proteins from rice exhibited closer relationships to maize and formed a monocot clade. EuAP2 in dicot and monocot plants formed a larger clade together. The moss clustered into one independent clade. These results suggested that euAP2 protein may be highly conserved in monocot and dicot and diverged after the split between bryophytes and vascular plants.

We also predicted the paralogous and orthologous relationships of the AP2 proteins among Arabidopsis, *B. napus*, *B. rapa* and *B. oleracea.* Seven pairs of paralogs were identified in *B. napus,* and 11 pairs of putative orthologous proteins were identified between species. To explore the driving force of gene divergence after gene duplication, the nonsynonymous/synonymous substitution ratio (Ka/Ks) of *euAP2* gene pairs was calculated (Table 2). With the exception of one gene pair (*BnaTOE1–1/BraTOE1–2*) with a Ka/Ks > 1, the majority of *euAP2* gene pairs had a Ka/Ks < 1, suggesting that the *AP2* gene family experienced strong purifying selective pressure during evolution.

**Table 2:** Ka and Ks values for orthologous and paralogous gene pairs between *B.napus* and another three plant species

	Gene Name	Gene Name	Ka	Ks	Ka/Ks	Selection pressure
Orthologous	*BnaAP2–2*	*BraAP2–2*	0.001	0	NA	NA
	*BnaAP2–4*	*BolAP2–2*	0	0	NA	NA
	*BnaSMZ-1*	*BraSMZ*	0	0	NA	NA
	*BnaSMZ-2*	*BolSMZ-2*	0.0139	0.0209	0.6651	Purifying selection
	*BnaSMZ-3*	*BolSMZ-1*	0	0	NA	NA
	*BnaTOE1–1*	*BraTOE1–2*	0.4165	0.3333	1.2496	Positive selection
	*BnaTOE1–3*	*BolTOE1–1*	0.0116	0.0243	0.4774	Purifying selection
	*BnaTOE2–2*	*BraTOE2–1*	0.0077	0.0424	0.1816	Purifying selection
	*BnaTOE2–3*	*BolTOE2–1*	0.001	0	NA	NA
	*BnaTOE3–1*	*BraTOE3*	0.0012	0	NA	NA
	*BnaTOE3–2*	*BolTOE3*	0	0	NA	NA
Paralogous	*BnaAP2–1*	*BnaAP2–5*	0.0076	0.0430	0.1767	Purifying selection
	*BnaAP2–2*	*BnaAP2–4*	0.0101	0.0485	0.2082	Purifying selection
	*BnaSMZ-1*	*BnaSMZ-3*	0.0128	0.0256	0.5000	Purifying selection
	*BnaTOE1–1*	*BnaTOE1–3*	0.0234	0.0716	0.3268	Purifying selection
	*BnaTOE1–2*	*BnaTOE1–4*	0.0078	0.0853	0.0914	Purifying selection
	*BnaTOE2–2*	*BnaTOE2–3*	0.0304	0.0943	0.3224	Purifying selection
	*BnaTOE3–1*	*BnaTOE3–2*	0.048	0.1088	0.4412	Purifying selection

We examined five types of gene duplications, namely, singleton, dispersed, proximal, tandem, and WGD or segmental duplication using the MCScanX program. No tandem repeat events within the *euAP2* gene family were found, while five dispersed and 11 segmental duplication events were identified (Table 3). These results indicated that some *euAP2* genes were probably generated by gene duplication and that the WGD or segmental duplication events were a major driving force of *euAP2* evolution [35].

**Table 3:** Duplication type of *euAP2* gene pairs

Gene ID	Gene Name	Gene ID	Gene Name	Duplication Type
BnaA01g34730D	*BnaAP2–1*			Dispersed
BnaA03g53830D	*BnaAP2–2*	BnaC01g01710D	*BnaAP2–3*	WGD or Segmental
BnaCnng39690D	*BnaAP2–4*			Dispersed
BnaCnng71740D	*BnaAP2–5*			Dispersed
BnaA09g54740D	*BnaSMZ-1*	BnaC08g25840D	*BnaSMZ-3*	WGD or Segmental
BnaC06g15540D	*BnaSMZ-2*	BnaC08g25840D	*BnaSMZ-3*	WGD or Segmental
BnaC02g15640D	*BnaSNZ*			Dispersed
BnaA03g22100D	*BnaTOE1–1*	BnaA07g13990D	*BnaTOE1–2*	WGD or Segmental
BnaA03g22100D	*BnaTOE1–1*	BnaC03g26480D	*BnaTOE1–3*	WGD or Segmental
BnaA03g22100D	*BnaTOE1–1*	BnaC04g15640D	*BnaTOE1–4*	WGD or Segmental
BnaA07g13990D	*BnaTOE1–2*	BnaC04g15640D	*BnaTOE1–4*	WGD or Segmental
BnaC09g13430D	*BnaTOE1–5*			Dispersed
BnaA02g06490D	*BnaTOE2–1*	BnaA10g12950D	*BnaTOE2–2*	WGD or Segmental
BnaA02g06490D	*BnaTOE2–1*	BnaC09g35430D	*BnaTOE2–3*	WGD or Segmental
BnaA10g12950D	*BnaTOE2–2*	BnaC09g35430D	*BnaTOE2–3*	WGD or Segmental
BnaA07g12050D	*BnaTOE3–1*	BnaC07g16190D	*BnaTOE3–2*	WGD or Segmental

Dispersed means that the gene might arise from transposition, such as ‘replicative transposition’, ‘non-replicative tranposition’ or ‘conservative transposition’;

WGD or segmental means that the gene might arise from Whole Genome Duplication or segmental duplication

**Fig. 3:**
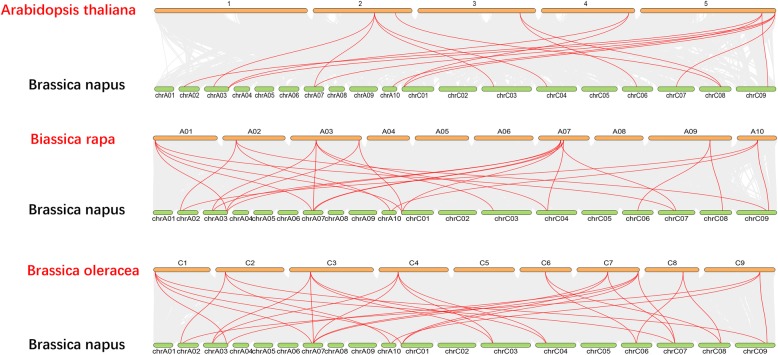
Synteny analysis of *euAP2* genes among *B. napus*, *Arabidopsis* and its ancestor parents. Grey lines in the background indicate the collinear blocks within the genomes of *B. napus* and other plants, and red lines highlight the syntenic *euAP2* gene pairs

To further infer the phylogenetic relationships of the *BnaAP2* family, we constructed three comparative syntenic maps of *B. napus* associated with Arabidopsis*, B. rapa* and *B. oleracea* (Fig. 3)*.* A total of 13 *BnAP2* genes showed a syntenic relationship with those in *Arabidopsis*, followed by *B. rapa* (13) and *B. oleracea* (13). Interestingly, collinear pairs (13 *BnAP2* genes) were identified between *B. napus* and the three other species, indicating that these orthologous pairs may have already existed before divergence (Additional file 2: Table S2).

### Structural organization and conserved domain analysis of *euAP2* genes

To gain further insights into the structure of the *euAP2* genes, we aligned their coding sequences to their genomic sequences (Additional file 9: Figure S2). The *euAP2* genes were classified into five subgroups. The difference in the number of exons (4 to 10), the intron length and the presence of an untranslated region (UTR) were assessed among the 19 genes, indicating that the *AP2*-type genes may perform different functions despite being in the same subfamily. However, the genes located at the end of the same branch always exhibited similar structures. The exon-intron structures were consistent with the phylogenetic relationships and conserved in each group, although some paralogous genes demonstrated some differences.

**Fig. 4:**
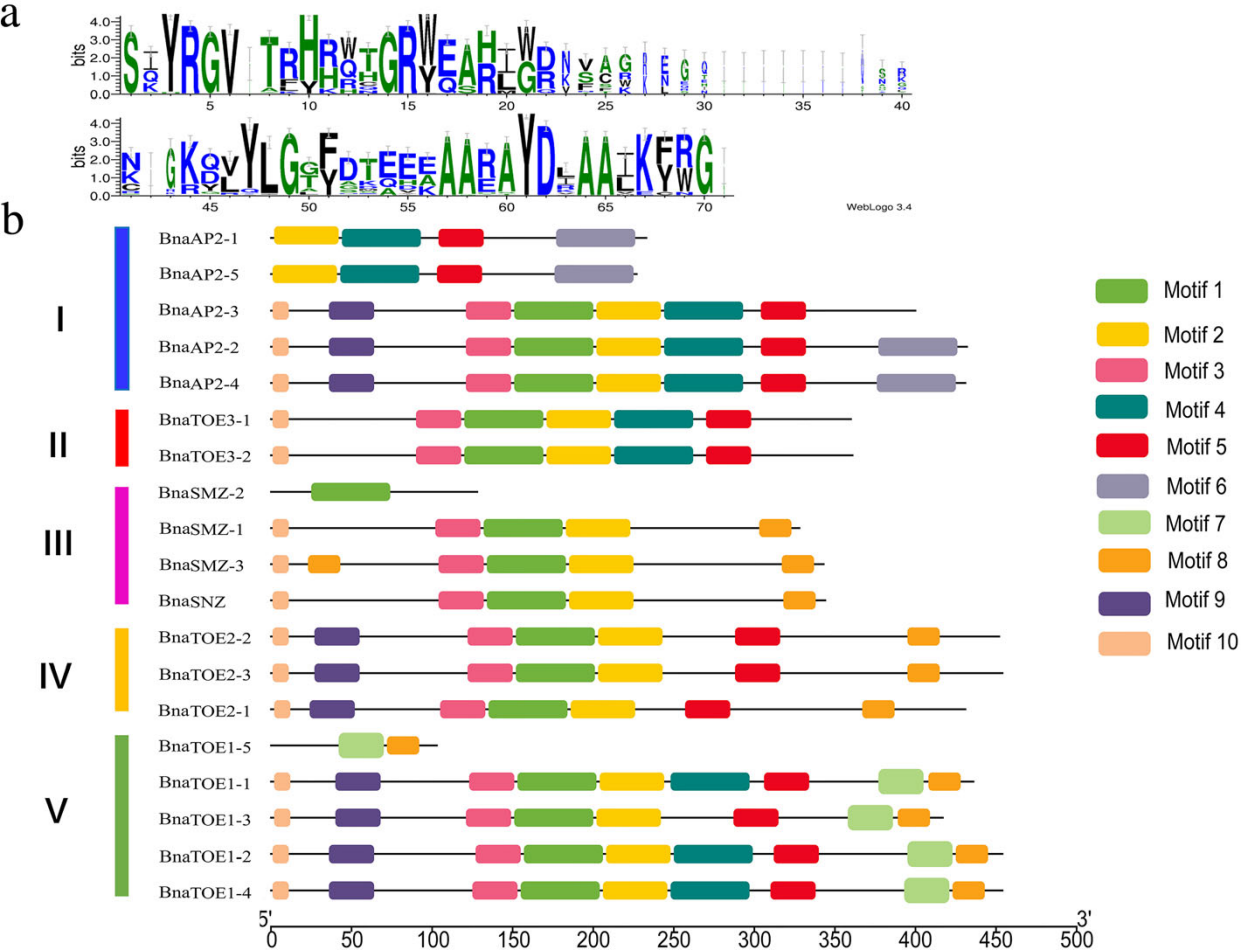
Organization of putative motifs in *euAP2* genes. **a** Sequence logo view of the consensus AP2 domain sequences in *B. napus.* The height of the letter (amino acid) at each position represents the degree of conservation. **b** Putative motifs in euAP2 protein identified by MEME. Numbered, coloured boxes represent different putative motifs. I to V indicate the phylogenetic subgroups from Additional file 9: Figure S2

MEME was used to identify putative conserved motifs among the *euAP2* genes (Fig. 4). The 10 most conserved motifs were recognized, and the length of the motifs ranged from 11 to 50 amino acids. The motif annotations are listed in Additional file 3: Table S3. Motifs 1 and 4 were annotated as the AP2-ERF domain, the other motifs had unknown functions. The conserved AP2 domain was created by WebLogo, and all the euAP2 proteins contain one or both of motifs 1 and 4, which further confirmed the identification results presented in Table 1. Most of the euAP2s within the same groups contained a similar motif composition. The similar motif number and arrangements among the AP2-type proteins in the same subgroups indicated that the protein function was conserved within a specific subfamily. However, there were also some exceptions: BnaAP2–1 and BnaAP2–5 had 4 motifs (motifs 2, 4, 5, and 6); BnaAP2–3 had 7 motifs; BnaAP2–2 / BnaAP2–4 contained 8 motifs; BnaSNZ had only one AP2-ERF motif; and BnaTOE1–5 had only 2 motifs, which was far fewer than the number observed in other proteins of the same subfamily (9 motifs). Motifs 6 and 7 were unique in Clades I and V, respectively, and may be important for the functions of BnaAP2 and BnaTOE proteins. These specific motifs and motif compositions may contribute to the functional divergence of *euAP2* genes.

In general, the similar gene structures and conserved motif compositions of euAP2 members in the same group as well as the results of the phylogenetic analysis, further confirm the reliability of the evolutionary classification.

### Analysis of cis-regulatory elements (CREs) of Bna-miR172 and *BnaAP2* genes

Temporal and spatial gene expression is regulated by the presence of different cis-regulatory elements in the promoters [36]. To understand the expression divergence, a 1000 bp region upstream of the transcription start site (TSS) of *euAP2* genes and a 1500 bp upstream of the Bna-miR172 precursor sequences were extracted to analyse CREs using the PlantCARE database (Additional file 4: Table S4). The identified CREs were classified into five main categories based on their function: light-responsive elements, hormone-responsive elements, stress-responsive elements, growth and development-responsive elements, and others. All the promoter sequences contained a TATA-box and CAAT-box. Light-responsive elements such as G-Box, GAG-motif, Box 4, AAAC-motif and Box 1 were abundant in most genes. CRE such as ERE, ABRE TGA element, GARE-motif, P-box, and CGTCA and TGACG motifs were responsive to various plant hormones including ethylene, abscisic acid, auxin, gibberellin and MeJA. Stress-responsive elements such as ARE, MBS, TC-rich repeats, LTR, Box-W1 and HSE have been reported in response to various abiotic stresses. In addition, other growth and development-responsive elements, such as the CCAAT-box, 5′ UTR Py-rich stretch and CCGTCC-box were found to be associated with endosperm and meristem expression and circadian control. In general, the expression of Bna-miR172 and its target *euAP2* genes could be regulated by diverse environments and internal developmental factors.

### Different expression profiles of *euAP2* genes

#### Gene expression patterns in different tissues

**Fig. 5:**
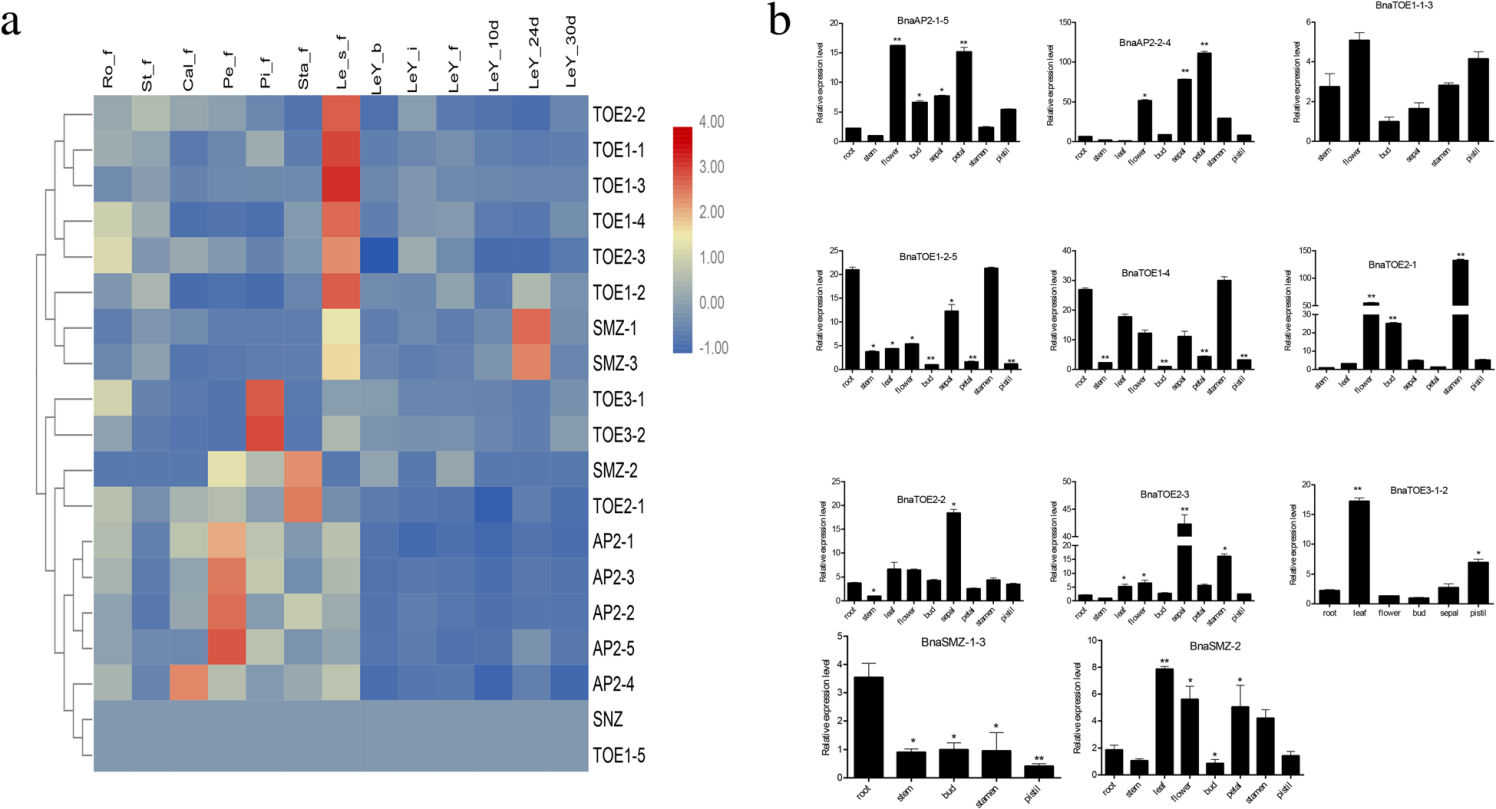
Expression profiles of *eu***AP2** genes in different vegetative and reproductive tissues of ZS11*.* **a** Hierarchical clustering of expression profiles of the eu***AP2*** genes in 13 samples based on the ZS11 RNA-Seq data. Heatmap showing the log2-transformed FPKM values*.* Tissues used for expression profiling are indicated at the top of each column. Every tissue has three replications. The genes are on the right of the expression bar. Ro-f, St-f, Cal-f, Pe-f, Pi-f and Sta-f represent root, stem, calyx, petal, pistil and stamen tissue collected from the full-bloom stage, respectively; Le_s_f, LeY-b, LeY-i, LeY-f, LeY-10d, LeY-24d and LeY-30d represent young leaves from the seedling stage, bud stage, early flowering stage, full-flowering stage, and 10 d, 24 d, and 30 d after flowering, respectively. **b** qRT–qPCR analysis of *eu****AP2*** genes in nine tissues. Root as a control. Data were normalized to the *actin* gene, and error bars indicate the standard error (SE) of the mean. Student’s *t*-test: *P < 0.05, **P < 0.01

To investigate the putative roles of *euAP2* genes, the expression patterns of all *euAP2s* were analysed in diverse tissues based on RNA-seq data (Fig. 5a). The *euAP2s* were classified into seven groups by using hierarchical clustering. The five *AP2* genes in the first group, i.e., *BnaTOE2–2*, *BnaTOE1–1*, *BnaTOE1–3*, *BnaTOE1–4* and *BnaTOE2–3*, were expressed at high levels in the young leaves from the seedling stage. The second group had three genes (*BnaTOE1–2*, *BnaSMZ-1*, and *BnaSMZ-3*) that were highly expressed in the young leaves from the seedling stage and 24 d after flowering. The transcription of *euAP2* genes in the third to sixth groups was enriched in the pistils, stamens, sepals and calyxes, which indicated their potentially specialized function in floral organ development. *BnaSNZ* and *BnaTOE1–5* in the seventh group showed almost no expression in these tissues.

qRT-PCR was used to confirm the expression levels of the miR172 target *euAP2* genes in nine tissues (root, stem, leaf, flower, bud, sepal, petal, stamen and pistil) of ZS11 (Fig. 5b). Most of the *euAP2*s exhibited a distinct tissue-specific expression pattern and achieved significant differences compared with root. *BnaTOE1–2-5* and *BnaTOE1–4* displayed the highest transcript abundance in the root and stamen; *BnaTOE3–1-2* and *BnaSMZ-1-3* exhibited the highest expression in the leaf and root, respectively; *BnaTOE2–2* and *BnaTOE2–3* were specifically highly expressed in the sepal; and the other five *euAP2* genes (*BnaAP2–1-5*, *BnaAP2–2-4*, *BnaTOE1–1-3*, *BnaTOE2–1*, and *BnaSMZ-2*) exhibited increased expression in the floral organs (flower, bud, sepal, petal, stamen, and pistil), which indicating that these genes may be involved in the regulation of flowering time and floral organ development.

#### Gene expression patterns in early- and late-flowering lines

To investigate the putative *AP2* genes involved in flowering time regulation, the RNA-seq data from both early-flowering bulk material and late-flowering bulk material was extracted. First, the expression levels in the shoot tissues (S) and leaves (L) of early-flowering and late-flowering bulk materials at the vegetative stage were analysed (Additional file 10: Figure S3a). The *euAP2* expression patterns were divided into 3 categories between two tissues of the two lines. The first group showed no or low expression in the four samples and included *BnaSMZ-1*, *BnaSMZ-2*, *BnaSMZ-3*, *BnaTOE1–5* and *BnaTOE2–1*; the second group exhibited medium level expression and no obvious difference among the four samples and included *BnaTOE1–1*, *BnaTOE1–4, BnaTOE2–2*, *BnaTOE2–3, BnaAP2–2* and *BnaAP2–3;* and the third group exhibited relatively high expression in the four samples, *BnaAP2–1, BnaAP2–5, BnaTOE1–2* and *BnaTOE1–3* displayed higher expression in the late-flowering material than that in the early-flowering material in both shoot tissues (S) and leaves (L), which indicated their negative roles in flowering regulation, while *BnaAP2–4*, *BnaTOE3–1* and *BnaTOE3–2* did not show the above rules. Second, we further studied the RNA-seq data from juvenile leaves of the early-flowering material (18Z134) and late-flowering material (18Z88) at the vegetative and reproductive stages and obtained similar results (Additional file 10: Figure S3b). *BnaSMZ-1*, *BnaSMZ-2*, *BnaSMZ-3*, *BnaSNZ*, *BnaTOE1–1*, *BnaTOE1–5* and *BnaTOE2–1* belonged to the first group and showed no or lower expression; *BnaTOE1–3*, *BnaTOE2–2*, *BnaTOE2–3*, *BnaTOE3–1* and *BnaTOE3–2* belonged to the second group and maintained a high expression level but exhibited no obvious difference among the four samples; *BnaAP2* (*AP2–1*, *AP2–2*, *AP2–3*, *AP2–4*, and *AP2–5*), *BnaTOE1–2* and *BnaTOE1–4* belonged to the third group and exhibited elevated expression in the vegetative-stage leaves and a higher transcript abundance in the late-flowering material than in the early-flowering material, which further confirmed their negative regulatory roles.

**Fig. 6:**
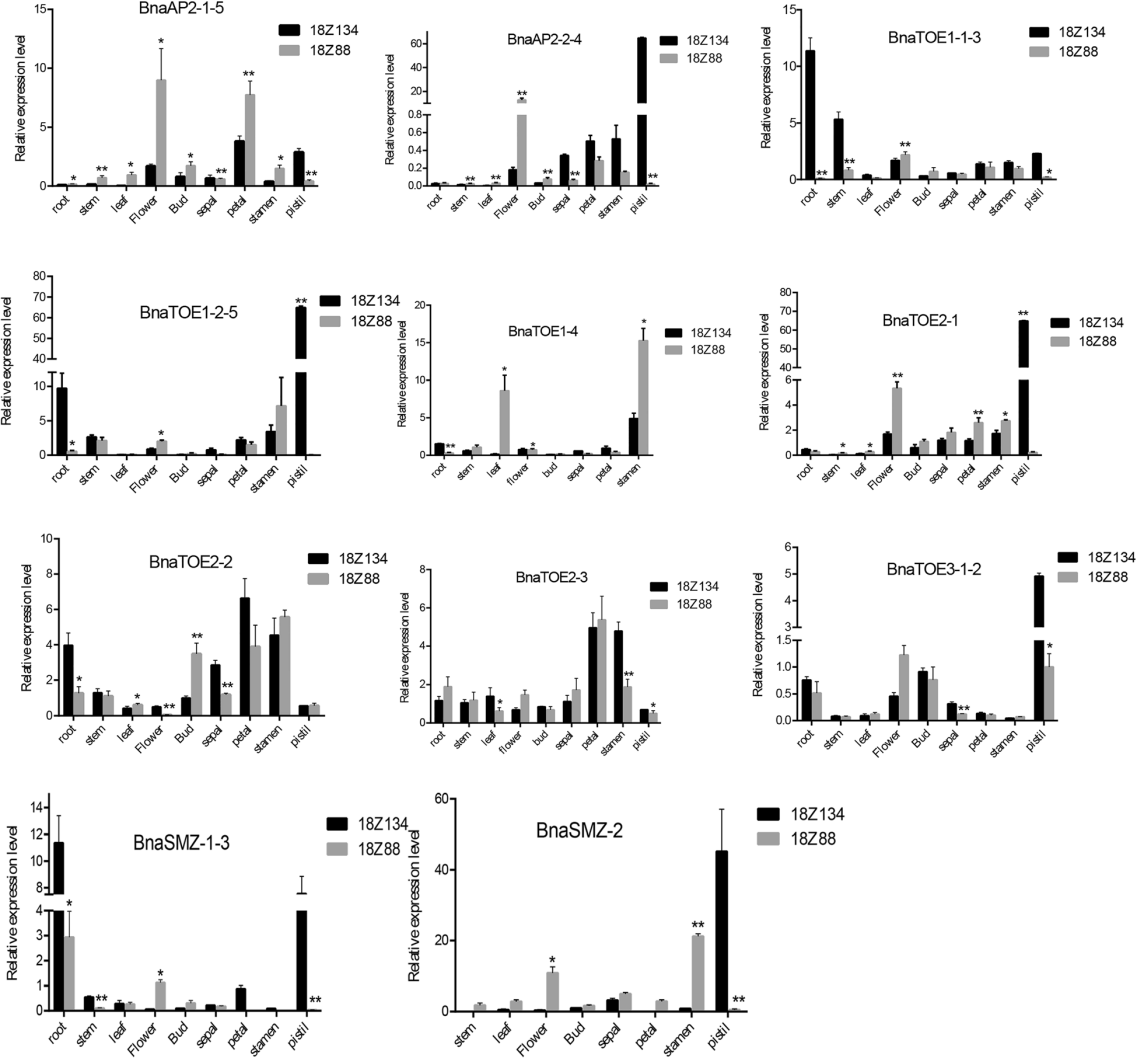
qRT–qPCR analysis of eu*AP2* genes in different tissues of early- (18Z134) and late-flowering (18Z88) lines. The error bars show standard error (SE) of three independent biological repeats. Student’s *t*-test: *P < 0.05, **P < 0.01

qRT-PCR was conducted to confirm the expression level of *AP2*s in the same materials (18Z88 and 18Z134) used for RNA-seq (Fig. 6). Most *euAP2* genes had higher expression levels in the floral organs of 18Z88 than 18Z134 and showed significant differences between them. A tissue-specific expression analysis revealed that *BnaTOE1–1-3*, *BnaTOE1–2-5*, *BnaTOE2–2* and *BnaSMZ-1-3* expressed abundantly in roots of 18Z134. All *euAP2* genes except *BnaTOE1–4* and *BnaTOE2–3* had higher expression levels in the pistil of 18Z134 than in 18Z88. The expression of *AP2*s in the other tissues presented relatively low expression in both lines.

## Discussion

### MiR172 and its target *AP2* genes in Brassica and their evolution

Transcription factors (TFs) play important roles in plant growth and development. The APETALA2/Ethylene Responsive Factor (AP2/ERF) proteins are a large family of TFs with a key function in response to biotic and abiotic stresses as well as developmental stages that have been identified in various plant species, including Arabidopsis [37], rice [38], *B. rapa* [39], *B. napus* [40, 41], barley and wheat [42], poplar [43] and soybean [44]. In this manuscript, we mainly focused on the *AP2* genes that belong to the *euAP2* group. The *euAP2* group in Arabidopsis includes six *AP2* genes (*AP2*, *TOE1*, *TOE2*, *TOE3*, *SMZ*, and *SNZ*). Each Arabidopsis *euAP2* gene has one to five orthologous genes in *B. napus*. In the present study, 19 *euAP2* genes were identified in *B. napus*, which is approximately more than twice the number identified in its progenitors *B. rapa* (8) and *B. oleracea* (9), indicating that some *euAP2* genes in *B. napus*, *B. rapa* and *B. oleracea* were subjected to a copy number increase due to WGD and polyploidization.

Based on the phylogenetic analysis, the *euAP2*s in *B. napus* can be classified into five groups (Additional file 9: Figure S2). Most *euAP2* genes in the same subgroup had similar exon-intron structures, although the exon number and intron length varied significantly among members of different subfamilies (Additional file 9: Figure S2). The majority of euAP2 proteins belonging to the same group contained similar motif distributions, while euAP2 proteins had distinct motif constitutions and organizations among subgroups (Fig. 4b), a finding that was also reported by Song [40]. The divergence in gene structure and motif composition between *euAP2* genes suggests functional differentiation during evolution. The motif recognition and gene structure analysis also highly corroborated our phylogenetic classification. Most miRNA target genes identified in plants are TFs [45]. MiR172 is known to regulate the *euAP2* group of AP2*-*like TFs through transcript cleavage and translational repression in Arabidopsis [3, 21, 22]*.* In this study, 4 members of the miR172 family in *B. napus* were identified, and target prediction showed that 17 of the 19 *euAP2* genes contained a MRE for miR172 (Fig. 1a; Table 1). Additionally, the miRNA172 binding site in the 3′ coding region were also identified in the *AP2* genes of barley and some representative euAP2 proteins [38, 42], suggesting that miR172 complementary sites in *euAP2* genes are conserved across plant species.

### Duplicated type and synteny analyses of *AP2* genes in *B. napus* and other species

Seven pairs of paralogs in *B. napus* and 11 pairs of orthologous proteins between species were identified via the phylogenetic analysis (Fig. 2b). Positive selection is the main driving force for functional divergence in euAP2 proteins after gene duplication. A comparison of the number of *euAP2* genes in *B. napus* with that in Arabidopsis and the ancestor parental genomes showed that *B. napus* has more genes (Fig. 3). Gene replication events that occurred during evolution contributed to the expansion of gene families [35]. Based on the duplication analysis, WGD or segmental duplication events had a major impact during *euAP2* evolution. Therefore, WGD or segmental duplication might be a possible reason for the increased number of *euAP2* genes.

### Analysis of the expression patterns of *euAP2*s in *B. napus*

The spatio-temporal expression patterns of *euAP2*s have been analysed in many species. Jofuku showed that, unlike other floral homeotic genes, *AP2* is expressed in both nonfloral (stems and leaves) and floral tissues (sepals, petals, stamens, carpels, developing ovules and inflorescence meristems) in Arabidopsis [46]. Javier studied the expression of *AP2*-like genes in the roots, stems, young leaves, and spikes at various developmental stages in wheat and barley and showed that *AP2*-like genes were expressed in all tissues, peaked in the early stages of spike development and gradually decreased with spike maturation [42]. The *euAP2* genes in rice were transcribed in the juvenile and mature leaves, root tip, inflorescences and serial stages of the panicles [38]. The expression of *AP2/ERFs* in *B. napus* was also previously examined: Zhuang and Zhu surveyed *B. napus* EST databases and showed that *AP2/ERF* genes were highly expressed in the stemsfollowed by flowers and leaves [47]; Hajar revealed that most of the *BnaAP2* subfamily showed high expression in the flower buds, roots, leaves, and stems via transcriptome sequencing [41]; Song used the RNA-seq data reported by Chahoub [32] and concluded that the *AP2* genes had higher gene expression in the roots than leaves [40].

Reports have indicated that the *AP2* genes have critical functions in flower ontogeny, including floral meristem establishment [27, 48–50], floral organ identity [3, 51, 52], and floral homeotic gene expression [44]. In this study, the *BnaAP2*s showed different expression patterns among tissues. More than half of the *euAP2* genes were expressed predominantly in reproductive organs such as the flowers, buds, sepals, petals, stamens, and pistils, suggesting that these *euAP2* genes also play important roles in floral development in *B. napus* [53].

To further validate whether the *euAP2* genes affect flowering time, we analysed their expression in different organs of early- and late-flowering lines. Certain *euAP2* genes, such as *BnaAP2–1*, *BnaAP2–5* and *BnaTOE1–2*, had higher expression levels in the floral organs of the late-flowering material (18Z88) than the early-flowering material (18Z134), indicating that they may be floral suppressor genes. This observation was consistent with the findings of previous studies in Arabidopsis [21, 22, 27].

## Conclusion

In this study, 19 *euAP2* genes and four miR172 members were identified in *B. napus* genome and the 19 *euAP2*s were categorized into five subgroups. The 17 euAP2 genes, which were predicted to be targeted by miR172, have tissue specific expression patterns. Most of the *euAP2*s were highly expressed in the floral organs (sepals, petals, stamens, and pistils), suggesting that the AP2/miR172 module plays an important role in flower organ development. In addition, three genes, *BnaAP2–1*, *BnaAP2–5* and *BnaTOE1–2*, had higher expression levels in late-flowering lines than in early-flowering lines*,* which revealed that they might act as floral suppressors. Taken together, the identification and expression of miR172/euAP2 genes provide insights for further research on the miR172/euAP2 interaction and flower development in *B. napus*.

## Materials and methods

### Identification of Bna-miR172 and its target *euAP2* genes

The nucleotide and protein sequences of the *AP2* family genes from *A. thaliana*, *B. napus*, *B. rapa*, and *B. oleracea* were downloaded from the Arabidopsis Information Resource (TAIR, http://www.arabidopsis.org) and BRAD (http://brassicadb.org/brad/index.php). Sequences from *A. lyrata*, rice, maize and moss were obtained from EnsemblePlants (http://plants.ensembl.org/index.html). BLASTP searches were performed against the BRAD and EnsemblePlants database with default parameters using Arabidopsis AP2 protein sequences as queries. Sequences with an E-value ≤10^− 10^ were regarded as candidate proteins. To further confirm the candidate *euAP2* family genes, the presence of the AP2 domain in the proteins was evaluated using the SMART (http://smart.embl-heidelberg.de/) [54] and Pfam (http://pfam.xfam.org/) [55] databases. To further validate the identified gene, the amino acid sequence of the identified genes were also submitted to the NCBI’s conserved domain database (CDD, https://www.ncbi.nlm.nih.gov/Structure/bwrpsb/bwrpsb.cgi) and HMMER (https://www.ebi.ac.uk/Tools/hmmer/search/hmmscan).

Physico-chemical parameters, including the theoretical isoelectric point (pI) and molecular weight (Mw) of the deduced proteins, were analysed using the ExPasy pI/Mw tool (http://web.expasy.org/protparam/) [56]. The subcellular localizations of the AP2 family proteins were predicted using the Cell-PLoc website (http://www.csbio.sjtu.edu.cn/bioinf/Cell-PLoc/).

The precursor and mature sequences of the plant miRNA172 family were obtained from the miRbase22 database (http://www.mirbase.org/) [57]. The miR172 genes were named as assigned in miRbase (Bna-miR172) and with new serial numbers (such as a-e). The secondary structures of the Bna-miR172 precursors were predicted by the Mfold web server (http://mfold.rna.albany.edu/?q=mfold) [58]. The putative target genes of miR172 were searched via the web-based tools TAPIR (http://bioinformatics.psb.ugent.be/webtools/tapir/) [59] and psRNATarget (http://plantgrn.noble.org/psRNATarget/) [60] by simultaneously uploading the miR172 mature sequence and *euAP2* gene sequence with default parameters. Finally, the potential targets predicted by both servers with one or two AP2 conserved domains were selected as candidate target genes.

Chromosome positional information for the *euAP2* and miR172 genes was investigated by using the *B. napus* database (http://www.genoscope.cns.fr/brassicanapus/). The MapChart version 2.2 was used to map the *euAP2* and Bna-miR172 genes [61].

### Phylogenetic and Ka/Ks analysis

Multiple sequence alignments of the protein sequences of the *euAP2* family genes and the precursor sequences of *miR172* were performed by ClustalW with default parameters [62]. The aligned sequences were then used to construct a neighbour-joining (NJ) tree by MEGA version 6.0 [63] with 1000 bootstrap replicates and pairwise deletion. The iTOL website (https://itol.embl.de/) was used to better visualize the phylogenetic tree [64]. The orthologous and paralogous relationships for *AP2* genes were inferred from the phylogenetic tree [65, 66]. The non-synonymous (Ka) and synonymous substitution rates (Ks) were calculated using DnaSp V6 software. Finally, the selection pressure experienced by gene pairs was assessed based on the Ka/Ks ratio [67].

### Duplicated type and synteny analyses of *euAP2*s in *B. napus* and other species

The Multiple Collinearity Scan toolkit (MCScanX) was adopted to analyse the gene duplication events, with the default parameters [68]. To demonstrate the synteny relationship of *AP2* genes obtained from *B. napus* and the three other species, syntenic analysis maps were constructed using Dual Synteny Plotter software (https://github.com/CJ-Chen/TBtools) [69].

### Analysis of exon–intron structure and motifs of *euAP2* genes

The exon-intron structures of the Brassica *euAP2* family genes were obtained by aligning coding sequences (CDSs) with their corresponding genomic sequences. The diagrams of exon–intron structure were created using the online Gene Structure Display Server (GSDS 2.0: http://gsds.cbi.pku.edu.cn) [70].

The sequence logo of the euAP2 domains was plotted by WebLogo (http://weblogo.berkeley.edu/logo.cgi). A conserved motif analysis within the determined euAP2 family was performed by the MEME program (http://meme-suite.org/) with the following parameters: optimum width, 6–300 amino acids; number of repetitions of a motif, any; and maximum number of motifs, 10 [71]. The motif sequence annotations were analysed by InterPro (https://www.ebi.ac.uk/interpro/beta/) [72].

### Analysis of cis-acting elements in the *B. napus* miR172 and *euAP2* gene promoters

The 1.5 kb upstream of the predicted transcription start site of Bna-miR172 precursor sequences and 1 kb upstream sequences of the euAP2 transcription start site were selected as the promoter regions. The intercepted promoter region was analysed for cis-regulatory elements using PlantCare (http://bioinformatics.psb.ugent.be/webtools/plantcare/html/) [73].

### Plant materials

The RNA-seq data were generated from 13 different tissues of the sequenced *B. napus* var. Zhongshuang 11 (ZS11) from the Oil Crop Research Institute, CAAS (root, stem, calyx, petal, pistil and stamen collected during the full-bloom stage, young leaves from the seedling stage, bud stage, early flowering stage, full-flowering stage, and 10 d, 24 d, and 30 d after flowering) (BioProject ID, PRJNA358784). The extremely early- and late-flowering materials were selected from the recombinant inbred line (RIL) constructed by our laboratory [74]. The flowering time of them were listed in Additional file 5: Table S5. The expression data from shoot tissues (S) and leaves (L) of early-flowering bulk materials (18Z43, 18Z71 and 18Z134) and late-flowering bulk materials (18Z44, 18Z88 and 18Z163) at the vegetative stage were collected, and the accession number was SRP108958. In addition, RNA-seq data from the juvenile leaves of early-flowering material (18Z134) and late-flowering material (18Z88) at the vegetative and reproductive stages were also analysed and the accession number was PRJNA540020. Two independent biological replicates were analyzed per sample. The transcript abundance of the *euAP2* genes was calculated using fragments per kilobase of exon model per million mapped reads (FPKM). The log2-transformed FPKM values were used to study *euAP2* gene expression (Additional file 6: Table S6).

### Tissue-specific expression analysis

ZS11, 18Z134 and 18Z88 were used for the expression analysis. Five vegetative tissues (root, stem, leaf, flower, and bud) and four floral organs (sepal, petal, stamen and pistil) were collected at the same developmental stage for the qRT-PCR. All plant materials were planted in the experimental field of Southwest University, Chongqing, China.

Total RNA was extracted from all samples with an RNAeasy extraction kit (Invitrogen, Carlsbad, CA, USA) according to the manufacturer’s instructions. The quality of RNA was assessed using an Agilent 2100 Bioanalyzer (Agilent, Böblingen, Germany) and examined through electrophoresis on a 1.5% agarose gel. For qRT-PCR, 1 μg of RNA was reverse transcribed into first-strand cDNA using a SuperScript™ First-Strand Synthesis System III (Invitrogen). Quantitative real-time PCR was performed with SYBR Green PCR Supermix on a real-time PCR machine (Bio-Rad, Hercules, CA, USA). The relative gene expression levels were calculated using the 2^−ΔΔCt^ method, with *BnaACTIN7* used as an internal control [75]. The qPCR primers are listed in Additional file 7: Table S7. At least two biological replicates were used, with three technical replicates performed for each sample.

## Additional files

Additional file 1 : **Table S1** The miR172 precursor and mature sequences identified in the *B.napus* genome. (XLSX 9 kb)

Additional file 2 : **Table S2** Collinear *euAP2* gene pairs among *B. napus*, *Arabidopsis* and its ancestor parents (*B.rapa* and *B. oleracea)*. (XLSX 12 kb)

Additional file 3 : **Table S3** Annotation of putative motifs of euAP2 proteins identified by MEME (XLSX 9 kb)

Additional file 4 : **Table S4** Cis-regulatory elements predicted in the promoter region of Bna-miR172 and *euAP2* genes (XLSX 11 kb)

Additional file 5 : **Table S5** Flowering time of extreme material in 5 years of Chongqing (XLSX 9 kb)

Additional file 6 : **Table S6** RNA-seq data of *euAP2* genes in various tissues of ZS11 and in leaf and shoot tissues of early- and late-flowering lines (XLSX 15 kb)

Additional file 7 : **Table S7** Primers used for qRT-PCR of *euAP2* genes in *B.napus (XLSX 10 kb)*

Additional file 8 : **Figure S1** Multiple sequence alignment of the bna-miR172 mature sequence and precursor sequence and its secondary structures. **a** Multiple sequence alignment of the bna-miR172 mature sequence; **b** Multiple sequence alignment of the bna-miR172 precursor sequence; **c** Secondary structures of the pre-miR172 sequence in *B. napus*. (TIF 4456 kb)

Additional file 9 : **Figure S2** Phylogenetic tree and gene structure of *euAP2* genes in *B. napus*. Subtree branch lines are coloured to indicate different clades. Blue boxes indicate untranslated 5′ and 3′ regions; yellow boxes indicate exons; black lines indicate introns. CDS, coding sequence; UTR, untranslated region. (TIF 1769 kb)

Additional file 10 : **Figure S3** Expression profiles of eu*AP2* genes in the early- and late-flowering lines based on RNA-Seq data. Hierarchical clustering results are shown on the left of the heat map and the coloured scale bar on the right side of the map represents log2-transformed FPKM values. Every sample has two replications. **a** Expression profiles of eu*AP2* genes in leaf and shoot tissues. EL, leaves of early-flowering material; LL, leaves of late-flowering material; ES, shoot apical regions of early-flowering material; and LS, shoot apical regions of late-flowering material. **b** Expression profiles of eu*AP2* genes in leaves at the vegetative and reproductive stages. EV, leaves of early-flowering material at the vegetative stage; LV, leaves of late-flowering material at the vegetative stage; ER, leaves of early-flowering material at the reproductive stage; and LR, leaves of late-flowering material at the reproductive stage. (TIF 2402 kb)

## Data Availability

All supporting data can be found within the manuscript and its additional supporting files.

## Abbreviations

*AP2*: *APETALA2*
CRE: Cis-regulatory elements
FPKM: Fragments per kilobase of exon per million fragments mapped
Ka: The ratio of the number of nonsynonymous substitutions per non-synonymous site
Ka/Ks: The nonsynonymous/synonymous substitution ratio
Ks: The ratio of the number of synonymous substitutions per synonymous site
MRE: MiRNA response element
qRT-PCR: quantitative real-time polymerase chain reaction
*SMZ*: *SCHLAFMUTZE*
*SNZ*: *SCHNARCHZAPFEN*
*TOE*: *TARGET OF EAT 1*
WGD: whole genome duplication
