# Supplementary figures and images for: Genome-wide exploration and characterization of miR172/euAP2 genes in Brassica napus L. for likely role in flower organ development
Source: BMC Plant Biol. 2019 Aug 1;19:336. doi: 10.1186/s12870-019-1936-2 (PMC6676641; doi:10.1186/s12870-019-1936-2)

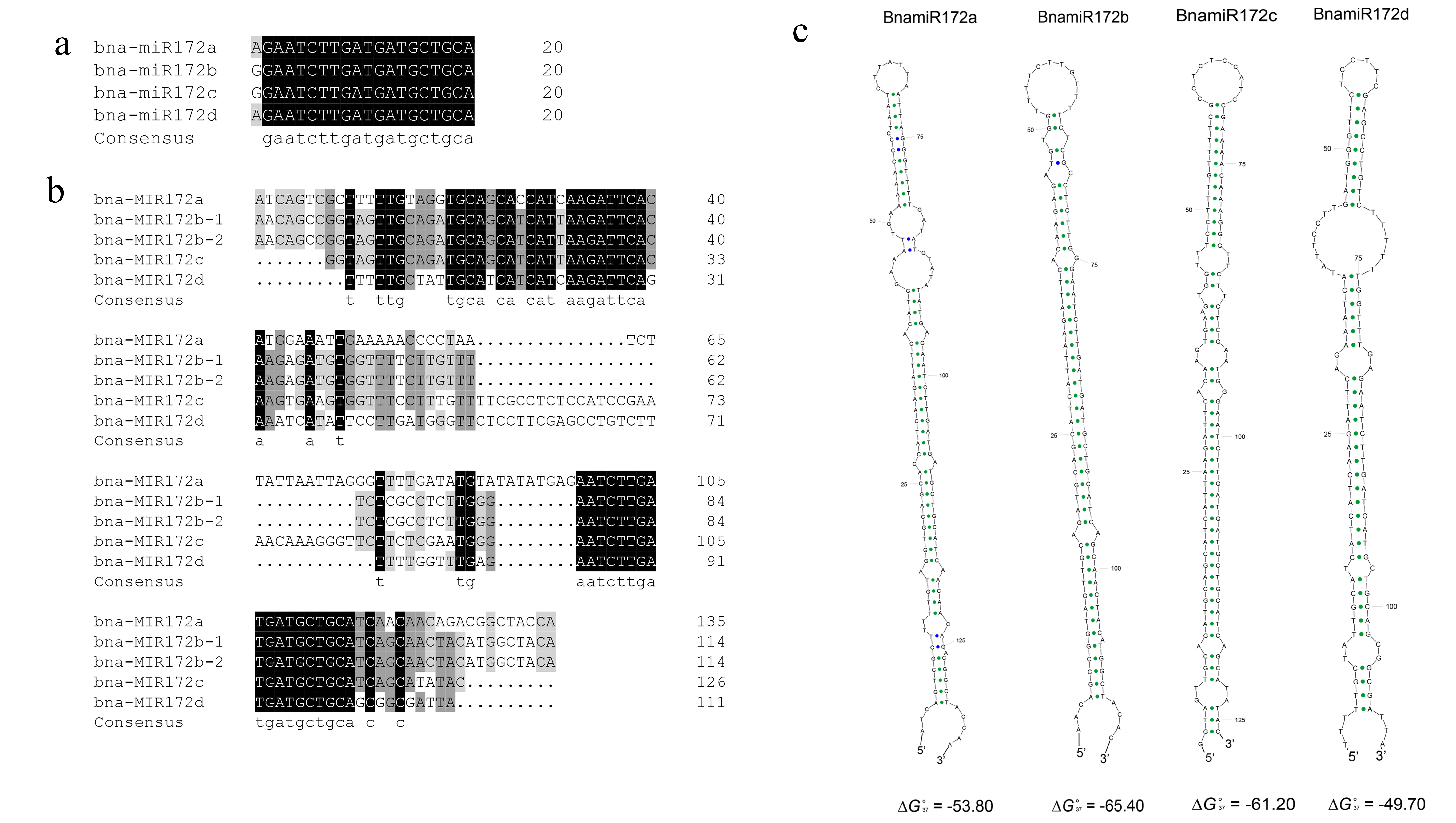

Supplement: Supplementary file 8 — : Figure S1 Multiple sequence alignment of the bna-miR172 mature sequence and precursor sequence and its secondary structures. a Multiple sequence alignment of the bna-miR172 mature sequence; b Multiple sequence alignment of the bna-miR172 precursor sequence; c Secondary structures of the pre-miR172 sequence in B. napus. (TIF 4456 kb) [file 12870_2019_1936_MOESM8_ESM.tif]

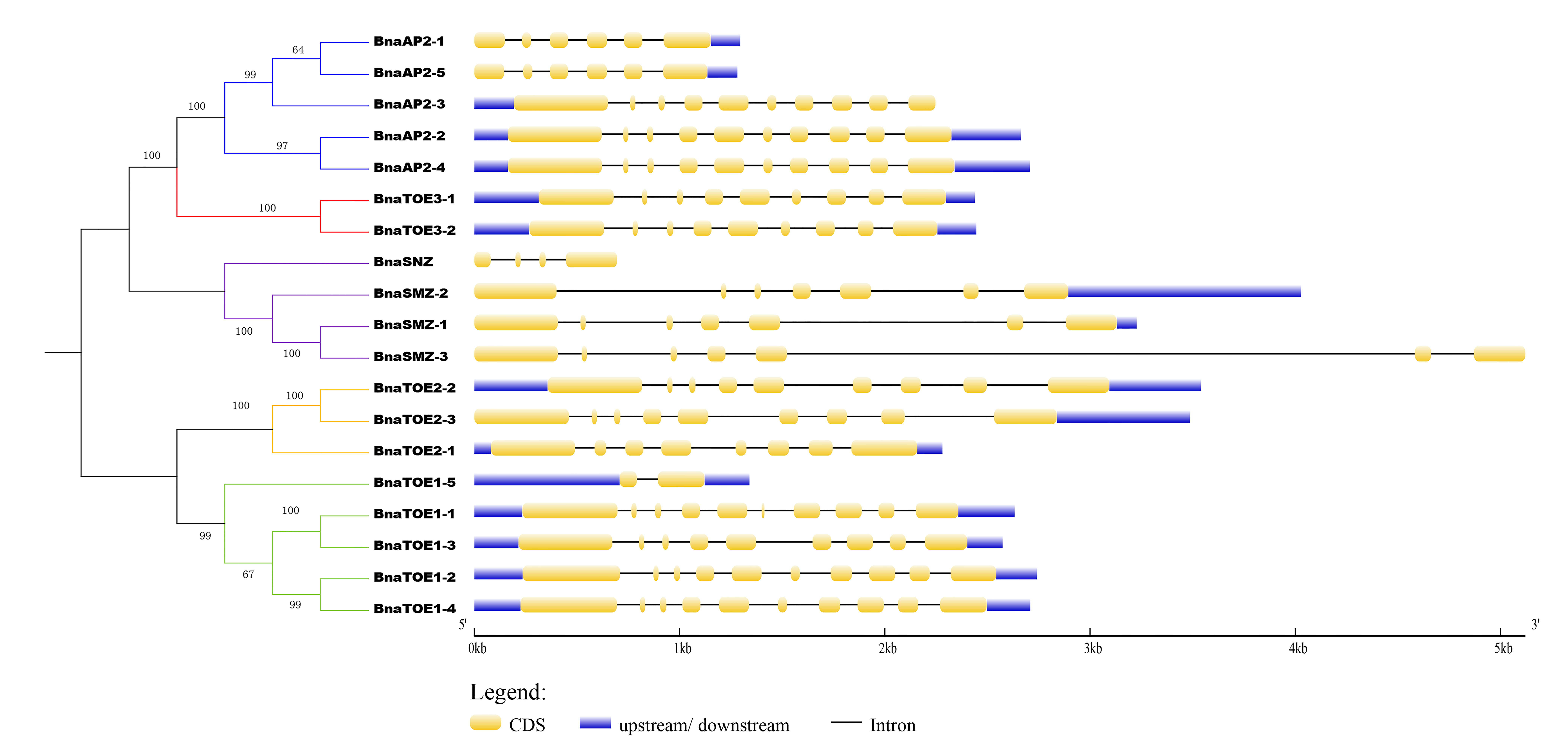

Supplement: Supplementary file 9 — : Figure S2 Phylogenetic tree and gene structure of euAP2 genes in B. napus. Subtree branch lines are coloured to indicate different clades. Blue boxes indicate untranslated 5′ and 3′ regions; yellow boxes indicate exons; black lines indicate introns. CDS, coding sequence; UTR, untranslated region. (TIF 1769 kb) [file 12870_2019_1936_MOESM9_ESM.tif]

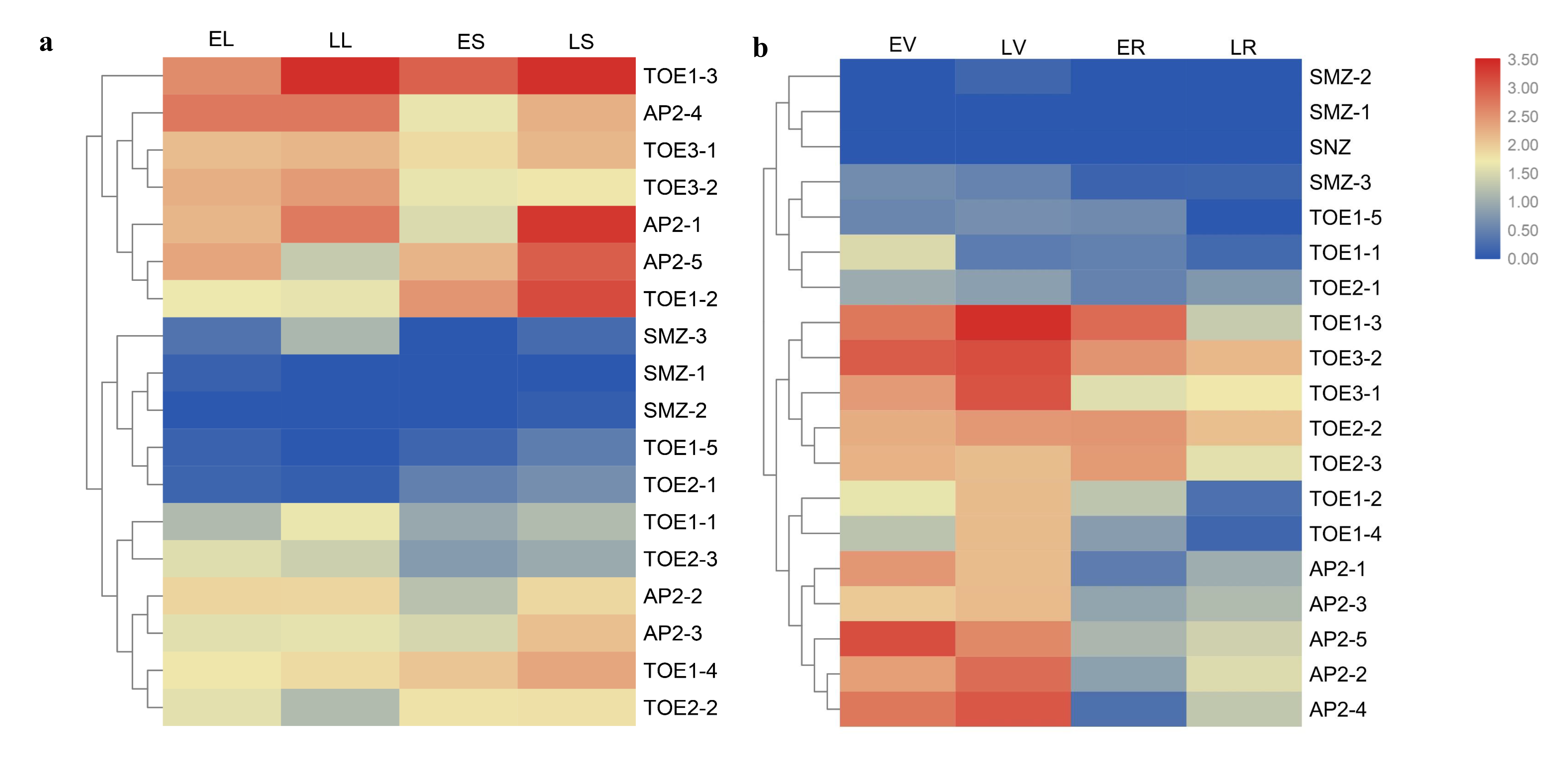

Supplement: Supplementary file 10 — : Figure S3 Expression profiles of euAP2 genes in the early- and late-flowering lines based on RNA-Seq data. Hierarchical clustering results are shown on the left of the heat map and the coloured scale bar on the right side of the map represents log2-transformed FPKM values. Every sample has two replications. a Expression profiles of euAP2 genes in leaf and shoot tissues. EL, leaves of early-flowering material; LL, leaves of late-flowering material; ES, shoot apical regions of early-flowering material; and LS, shoot apical regions of late-flowering material. b Expression profiles of euAP2 genes in leaves at the vegetative and reproductive stages. EV, leaves of early-flowering material at the vegetative stage; LV, leaves of late-flowering material at the vegetative stage; ER, leaves of early-flowering material at the reproductive stage; and LR, leaves of late-flowering material at the reproductive stage. (TIF 2402 kb) [file 12870_2019_1936_MOESM10_ESM.tif]
